# Crosslinking stabilization strategy: A novel approach to cartilage-like repair of annulus fibrosus (AF) defects

**DOI:** 10.1016/j.mtbio.2025.101625

**Published:** 2025-03-05

**Authors:** Zihan Wang, Lei Wang, Shaoshuo Li, Xin Chen, Bo Chen, Zhichao Lou, Zheng Li, Rongrong Deng, Lin Xie, Jianwei Wang, Xin Liu, Ran Kang

**Affiliations:** a Affiliated Hospital of Integrated Traditional Chinese and Western Medicine, Nanjing University of Chinese Medicine, Jiangsu Province, 210028, PR China; b Wuxi Hospital Affiliated to Nanjing University of Chinese Medicine, Jiangsu Province, 214000, PR China; c Department of Orthopedics, Nanjing Lishui Hospital of Traditional Chinese Medicine, Nanjing, Jiangsu Province, 210028, PR China; d The Third Clinical Medical College, Nanjing University of Chinese Medicine, Nanjing, Jiangsu Province, 210028, PR China; e College of Materials Science and Engineering, Nanjing Forestry University, Nanjing, Jiangsu Province, 210037, PR China; f Peking Union Medical College Hospital, Beijing, 100730, PR China; g Materials Science and Devices Institute, Suzhou University of Science and Technology, Suzhou, 215009, PR China

**Keywords:** Cartilage repair, Stress stimulation, Annulus fibrosus (AF), Fibrin-genipin adhesive hydrogel, Mesenchymal stromal cells (MSCs), RhoA/ROCK

## Abstract

Lumbar disc degeneration due to annulus fibrosus (AF) defects poses a significant challenge in clinical treatment Current treatments exhibit limited repair efficacy and a high recurrence rate. To address this, we devised a novel approach of crosslinking stabilization strategy. We integrated fibrinogen, thrombin, genipin, and human bone marrow-derived mesenchymal stem cells (hBMSCs) hydrogel (FTGB) with acellular scaffold and fascia (FTGB@S@F) to remediate AF defects. FTIR analysis confirmed stable chemical crosslinking within the FTGB hydrogel. FTGB hydrogel demonstrated superior biocompatibility compared to the FB hydrogel, with significantly higher cell viability (97.60 ± 2.02 % *vs* 81.43 ± 4.50 %, P < 0.01) and enhanced proliferation and migration, as shown in DAPI, Edu and phalloidin staining. Atomic force microscopy (AFM) revealed that FTGB@S has a dense reticular structure, enhancing material performance with higher elastic modulus than FB@S. MTS testing showed that FTGB@S@F outperformed other groups in resisting cyclic axial load (25.53 ± 1.17 MPa) and maintaining disc height (0.57 ± 0.12 mm), with stable axial compression resistance and minimal deformation. It also exhibited the lowest rupture ROM (1.45 ± 0.17 mm) and a rupture modulus close to the Intact control, demonstrating its potential to restore AF mechanical function. MRI imaging revealed that the FTGB@S@F group preserved an intact AF structure with high signal intensity, a significantly larger NP area (223.64 ± 73.32 mm^2^*vs* 137.30 ± 75.31 mm^2^, P < 0.05), and higher disc height (102.5 ± 73.32 % *vs* 88.50 ± 12.86 %, P < 0.05). Histology confirmed superior AF repair and reduced NP degeneration in the FTGB@S@F group compared to the Un-repair and FB@S@F groups. Transcriptomic analysis identified upregulation of PIGR and downregulation of COL4A3, linked to the PI3K-Akt pathway. Immunohistochemical and qPCR analyses showed enhanced expression of COL1, Aggrecan, and RhoA, indicating effective regeneration.

## Graphical abstract

Figure. Schematic diagram of the crosslinking stabilization strategy and mechanism diagram of the crosslinking stabilization strategy promoting the differentiation of MSCs into fibrocycle-like cells.

**Figure:**
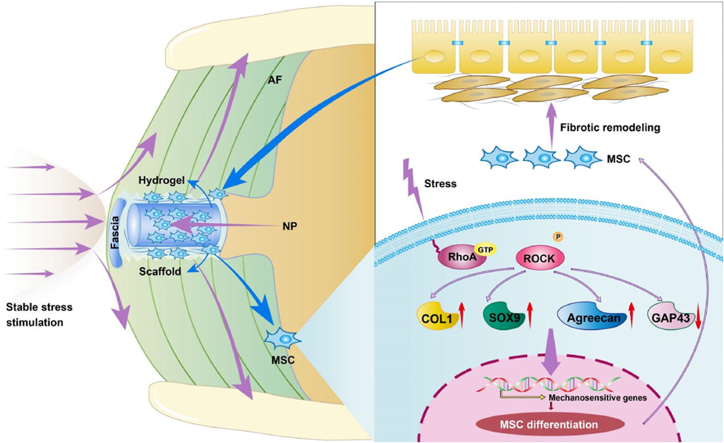

## 1 Introduction

**Fig. 1:**
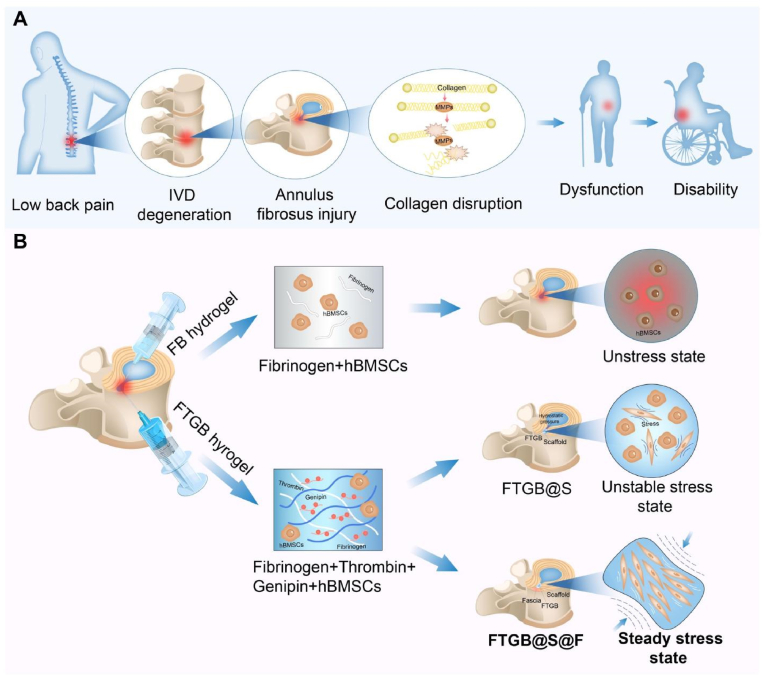
Mechanisms of FTGB@S@F in preventing the progression of low back pain. (A) Schematic diagram of pathogenesis of low back pain. (B) Schematic diagram of the critical role of synergistic internal and external mechanical stability in driving the orderly differentiation of stem cells.

Low back pain is a common and debilitating condition, affecting approximately 23 % of the global population, with a recurrence rate of 24–80 % within one year [1]. It is often caused by intervertebral disc (IVD) degeneration due to annulus fibrosus (AF) injury, which impairs the biomechanical stability of the spine [2]. The annulus fibrosus (AF), as the outer structure of the intervertebral disc (IVD), is composed of 15–25 concentric lamellae of collagen fibers, with fibers in each layer aligned in one direction and alternating with the adjacent layers. This unique architecture enables the AF to resist multidirectional tensile, bending, and torsional stresses, maintaining the mechanical stability of the entire spine. Once the AF is damaged, local inflammatory responses alter its composition, compromising its mechanical properties [3]. These changes lead to uneven stress distribution, causing an imbalance in the loading of collagen fibers, which in turn alters the spatial structure of collagen monomers and their triple helix. This exposes cleavage sites, where matrix metalloproteinases (MMPs) bind and degrade underloaded collagen fibers, further disrupting the AF structure [4]. This cascade of events exacerbates the biomechanical instability of the AF, accelerating AF damage and IVD degeneration (Fig. 1A). Current treatments for degenerative disc disease, such as conservative therapy and surgery, primarily alleviate symptoms without addressing the root cause of degeneration, namely AF injury.

The development of regenerative medicine and tissue engineering offers promising strategies to address these challenges. Among the various approaches, stem cell therapy, hydrogel technology, and tissue engineering scaffolds are key components in advancing AF repair. Stem cells, particularly human bone marrow-derived mesenchymal stem cells (hBMSCs), have demonstrated potential in AF regeneration by secreting cytokines and growth factors that support tissue repair [5,6]. However, effectively inducing hBMSCs to differentiate into AF-like cells remains challenging, largely depending on mechanical stimulation and intrinsic cytokine signaling [7,8]. Scaffolds play a crucial role in this process, providing both mechanical support and a vehicle for seeded cells [9,10]. An ideal scaffold material must meet several requirements, including appropriate mechanical properties, biocompatibility, biodegradability, and ease of delivery. Although synthetic materials are easily manufactured and modified, they often fail to effectively mimic the native IVD microenvironment and its complex mechanical properties, limiting their effectiveness in promoting tissue regeneration [11]. Decellularized matrices have emerged as a potential alternative, as they retain the native extracellular matrix (ECM) structure and mechanical properties while removing immunogenic cells, thereby providing an environment similar to the original tissue for seeded cell growth and differentiation [12].

Hydrogels can help stabilize stress distribution and provide an ECM-like environment that promotes cell migration and integration [13]. To be effective, hydrogels must exhibit high adhesive strength, low cytotoxicity, and high failure strain to prevent stress concentration and mechanical failure[[14], [15], [16]]. However, the mechanical strength of natural hydrogels is often limited, leading to the development of crosslinked hydrogels with other materials, such as genipin extracted from Gardenia [17,18]. Genipin has low toxicity and high biostability, and it has been shown to enhance the mechanical properties of the IVD. Genipin-crosslinked hydrogels can partially restore the compressive and shear properties of the AF and improve the overall biomechanical function of the spinal unit [19].

Mechanical stimulation plays a crucial role in annulus fibrosus (AF) repair, with the RhoA/ROCK signaling pathway being a key mechanism in this process [20]. This pathway converts external mechanical signals into biochemical cues that regulate cytoskeletal organization and cellular behavior [21]. When hBMSCs are subjected to mechanical stress, such as compression or tension, the RhoA/ROCK pathway is activated, promoting actin polymerization and cytoskeletal reorganization, enhancing cellular contractility and mechanical stability [22,23]. This supports hBMSCs differentiation into AF-like cells. The cytoskeletal components, including stress fibers, microtubules, and intermediate filaments, work together under the regulation of the RhoA/ROCK pathway to sense and transmit mechanical forces, further aiding tissue repair and adaptation [24]. Therefore, modulating the RhoA/ROCK pathway through mechanical stimulation not only enhances AF regeneration but also improves its biomechanical properties.

Based on these findings, we propose a crosslinking stabilization strategy to restore the mechanical conduction pathways and biomechanical function of damaged AF tissue while promoting orderly regeneration (Fig. 1B). We integrated fibrinogen, thrombin, genipin, and hBMSCs hydrogel (FTGB) with acellular scaffold and fascia (FTGB@S@F) to remediate AF defects (Table 1). To assess the safety and efficacy of this approach and to highlight the critical role of stable mechanical stress in AF regeneration, we conducted a comprehensive series of *in vitro* and *in vivo* experiments. These included evaluations of biocompatibility, mechanical properties, MRI imaging, immunohistochemical staining, transcriptomic analysis, and qPCR. The aim was not only to validate the effectiveness of our proposed strategy but also to deepen our understanding of how mechanical stimulation contributes to AF regeneration and repair.

**Table 1:** Sample abbreviations for each combination of hydrogel composition, scaffold and fascia.

Samples	Abbreviations
Human bone marrow-derived mesencgymal stem cells	hBMSCs
Fibrinogen (Fib) + hBMSCs	Fib + hBMSCs (FB)
Fibrinogen + Thrombin + Genipin	FTG
Fibrinogen + Thrombin + Genipin + hBMSCs	Fib-T-G + hBMSCs (FTGB)
Fib + hBMSCs + Scaffold	FB@S
Fib-T-G + hBMSCs + Scaffold	FTGB@S
Fib + hBMSCs + Scaffold + Fascia	FB@S@F
Fib-T-G + hBMSCs + Scaffold + Fascia	FTGB@S@F

## 2 Materials and methods

### 2.1 Preparation of the FTGB and FB hydrogels

**Fig. 2:**
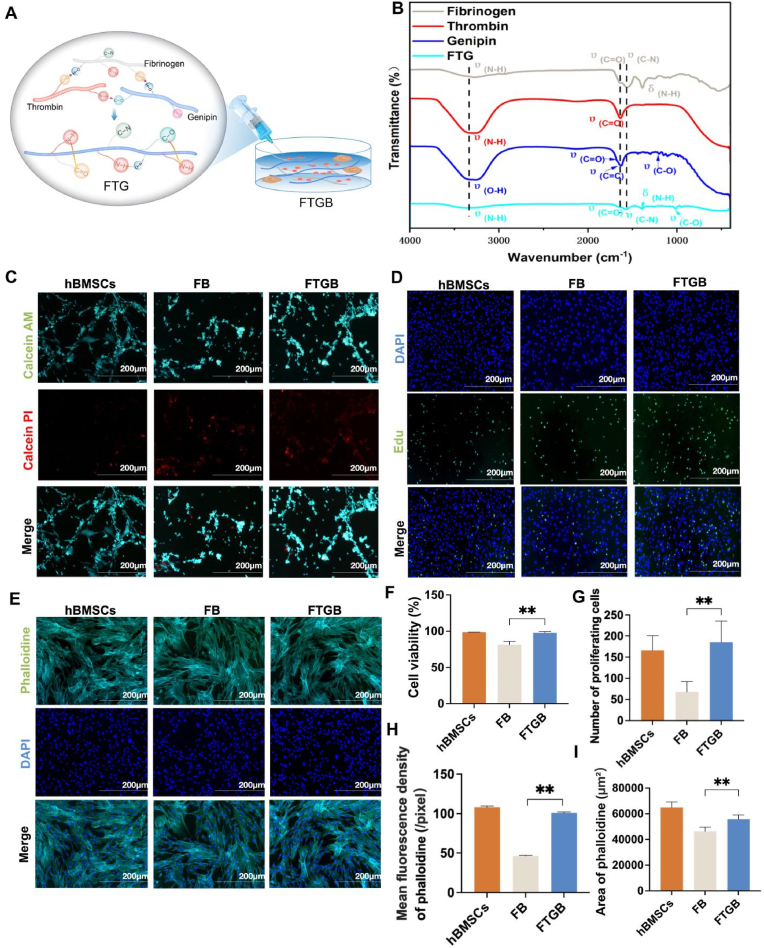
Characterization and biocompatibility test. (A) Schematic illustration of the composition of Fibrinogen + hBMSCs (FB) and Fibrinogen + Thrombin + Genipin + hBMSCs (FTGB) hydrogels. (B) Fourier transform infrared (FTIR) spectra of fibrinogen, thrombin, genipin, and the FTG solution. (C) Calcein AM/PI staining showing live (green) and dead (red) hBMSCs cultured for 3 days on FB and FTGB hydrogels. (D) DAPI (blue) and EdU (green) staining of hBMSCs cultured for 3 days on FB and FTGB hydrogels. (E) Phalloidin (green) and DAPI (blue) staining of hBMSCs cultured for 3 days on FB and FTGB hydrogels. (F) Cell viability corresponding to Fig. 2C. (G) Quantification of proliferating cells corresponding to Fig. 2D. (H) Mean fluorescence intensity of phalloidin corresponding to Fig. 2E. (I) Area of phalloidine staining areas corresponding to Fig. 2E. (J) Mean fluorescence density in channel blue, green and red. The error bars indicate SD; n = 3, biological replicates. (∗P < 0.05 and ∗∗P < 0.01).

Human bone marrow-derived mesenchymal stem cells (hBMSCs) (7500, ScienCell, USA) were purchased for resuscitation, culture, and passage, and P6 generation with good culture growth was selected for the follow-up experiment. The FTGB hydrogel containing 140 mg/mL Fibrinogen (Fib, F3879, Sigma, USA), 28 U/mL thrombin (T4393, Sigma, USA), and 6 mg/mL genipin (G4796, Sigma, USA) was mixed with 5 × 10^4^/50 μl hBMSCs. The FB adhesive hydrogel had the same composition but lacked thrombin and genipin (Fig. 2A). The adhesive hydrogels were prepared in a sterile environment just before application.

### 2.2 Fabrication of acellular scaffold

Fresh bovine tails (buffalo, mixed gender, 28 months old) were received from an abattoir within 5 h of slaughter. AF samples harvested from fresh bovine tails were frozen in liquid nitrogen for 22 h before thawing in a hypotonic buffer (10 mM tris-HCl, pH 8.0) at 37 °C for 2 h. The AF samples were then pre-washed with 75 % ethanol for 2 h. Subsequently, the samples were incubated in the decellularization solution, which consisted of Tris-HCl buffer (10 mM Tris-HCl, pH 8.0) containing 0.2 % SDS, 0.1 % EDTA, and 10 KIU/mL aprotinin at 4 °C for 24 h with gentle agitation (40 r/min) on an orbital shaker. After transient rinsing, the samples were submerged in deoxyribonuclease (DNase 50 U/mL; Sigma, USA) and ribonuclease (RNase 1 U/mL; Sigma, USA) in Tris buffer (50 mM Tris-HCl, 10 mM magnesium chloride, and 50 mg/mL bovine serum albumin at pH 7.5) for 3 h at 37 °C with gentle agitation. The cellular scaffolds with good biocompatible were acquired after three washes in PBS for 8 h [25]. The scaffolds were freeze-dried and stored in a sterile environment for further use.

### 2.3 Fourier transform infrared spectrometer (FTIR)

The FTIR spectra of fibrinogen, thrombin, genipin and FTG solution were analyzed by fourier transform infrared spectrometer (FTIR, TENSOR 27, BRUKER, Germany) in the range of 4000 cm^−1^ to 400 cm^−1^ at a resolution of 4 cm^−1^ with 128 coadded scans. Spectral data was collected and processed by OPUSTM software.

### 2.4 Biocompatibility

The biocompatibility of hydrogels is critical for *in vivo* applications, so we used well-grown hBMSCs to evaluate their biocompatibility. The experiment was divided into three groups: Intact control group, FB group and FTGB group (n = 3, biological replicates). Next, Edu assay, Live/Dead cell staining, and cytoskeleton staining were performed:

*Edu assay*: The cell proliferation kit (C0071S, Beyotime, China) was used for Edu assay, and the cell culture plate was transwell plate. First, hBMSCs with a density of 2.5 × 10^4^ cells/well were inoculated in the lower chamber of the 24-well transwell plate for 3 d, followed by the addition of FTG and Fib hydrogels in the upper chamber, and the hydrogels were kept completely immersed in the cell culture medium and stabilized for 2 h. Then, the Edu was diluted with 1:500 cell culture solution to obtain 2X Edu working solution. Subsequently, 2X Edu working solution with the same volume as the protocell culture solution was added to the 24-well plate, the final Edu concentration was diluted to 1X, and the incubation continued for 2 h. Further, removed from the medium and washed the cells three times with PBS (pH 7.40, 0.01 M) for 3–5 min each time. Next, 200 μl 4 % paraformaldehyde fixing solution (16005, Sigma, USA) was added to each well and fixed at room temperature for 15 min. Then the fixing solution was removed, and the cells were washed three times with PBS for 3–5 min each time. Then the PBS solution was removed and 0.3 % Triton X-100 PBS solution (V900502, Sigma, USA) was added to it and incubated at room temperature for 10–15 min. The PBS solution was removed, and the cells were washed 1–2 times with PBS for 3–5 min each time. Then the PBS solution was removed, and 200 μl of Click reaction solution was added to it and incubated at room temperature away from light for 30 min. Then the Click reaction solution was removed, and the cells were washed 3 times with PBS for 3–5 min each time. Subsequently, further nuclear restaining was performed using DAPI (D9542, Sigma, USA) and incubated at room temperature in dark for 10 min. Then, DAPI was removed, and the cells were washed with PBS for 3 times for 3–5 min each time. Finally, the images were collected using an inverted fluorescence microscope, where green is the proliferating cell and blue is the nucleus. The number of proliferating cells and nucleus was calculated through ImageJ software (Version 2.3.0).

*Live/Dead cell staining*: The culture scheme of cells and hydrogels can refer to *Edu assay*. After the culture, the culture medium of each group was removed, washed with PBS, added with calcein AM/PI staining solution (KGAF001, KEYGEN BIOTECH, China), and incubated at room temperature for 30–45 min, and then observed with fluorescence microscope and collected images. Live cells are shown in green and dead cells in red. The number of live cells and dead cells was calculated by ImageJ software (Version 2.3.0). Cell viability was calculated using the following formula: $\text{Cell}\;\text{viability}\;\left(\%\right)=\frac{\text{Live}\;\text{cells}}{\text{Live}\;\text{cells}+\text{dead}\;\text{cells}}\times$ 100.

*Phalloidine staining*: Refer to *Edu assay* for cell and hydrogel culture protocols. After the end of culture, the culture medium of each group was removed, and the cells were washed with PBS three times for 3–5 min each time. Then, added 4 % paraformaldehyde fixing solution to each well, fixed at room temperature for 10 min, removed the fixing solution, and washed with PBS for three times, 3–5 min each time. Next, 0.5%Triton X-100 permeable solution was added to each well and applied for 5 min, then washed three times with PBS for 3–5 min each time. Then 200 μl of the prepared working solution of FITC-labeled phalloidin (CA1620; Solarbio, China) was added and incubated for 30 min at room temperature in the dark. It was then removed and washed three times with PBS for 3–5 min each time. Immediately after that, 200 μl of DAPI solution was added and nuclear staining was performed for 5 min. Finally, they were washed 2–3 times with PBS and photographed under an inverted fluorescence microscope. The green is for cytoskeleton staining and the blue is for nucleus staining. Area of phalloidine staining areas and mean fluorescence density were calculated in blue, green and red channels respectively by ImageJ software (Version 2.3.0).

### 2.5 Scanning electron microscope (SEM)

**Fig. 3:**
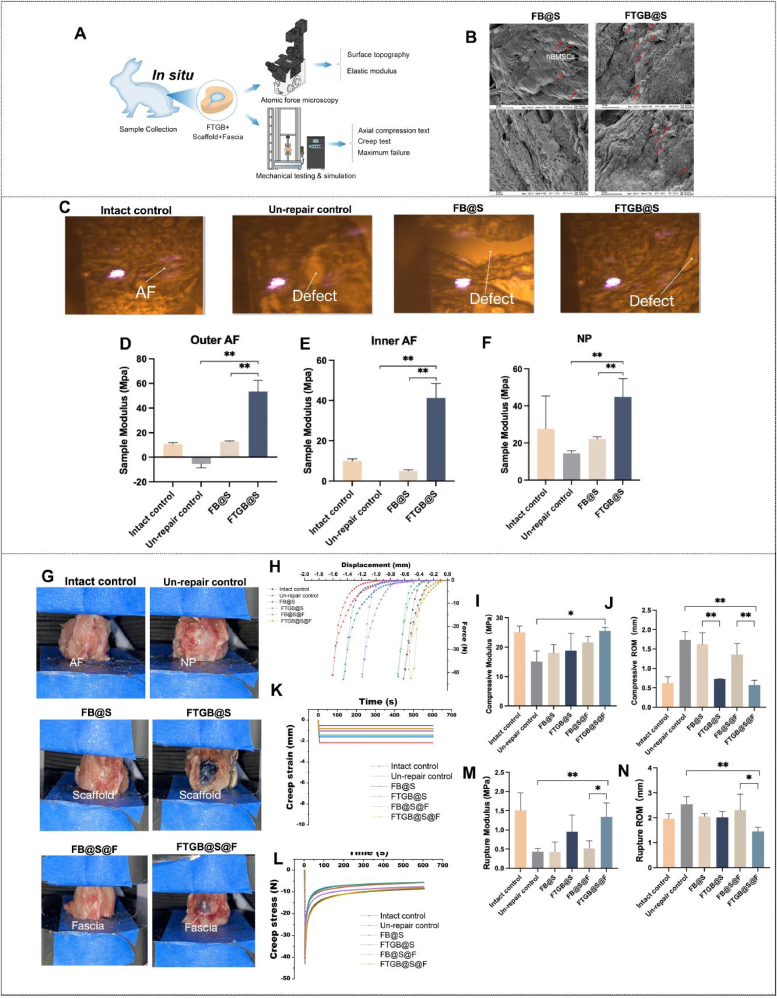
Nanoscale mechanical and biomechanical characterization. (A) Diagram of nanoscale mechanical and biomechanical characterization. (B) Scanning electron microscope (SEM) images of Fib + hBMSCs + Scaffold (FB@S) and Fib-T-G + hBMSCs + Scaffold (FTGB@S) (The red arrows refer to hBMSCs). (C) Microscopic morphology of the disc tissue defect observed under atomic force microscopy (AFM). (D–F) Elastic modulus at different positions of outer AF, inner AF, and NP. (G) Representative samples of Intact control, Un-repair control, FB@S, FTGB@S, Fib + hBMSCs + Scaffold + Fascia (FB@S@F) and Fib-T-G + hBMSCs + Scaffold + Fascia (FTGB@S@F). (H) Representative force-displacement curves in compression. (I) Compressive modulus. (J) Compressive ROM. (K) Creep stress. (L) Creep strain. (M) Rupture modulus. (N) Rupture ROM. The error bars indicate SD; n = 4, biological replicates. (∗P < 0.05 and ∗∗P < 0.01).

The prepared FB@S and FTGB@S were rinsed with saline, dried, and sterilized with ethylene oxide. All samples were then dehydrated at a gradient of 70%–80%-90%-95%-100 %. The surface morphology and internal structure of FB@S and FTGB@S were observed by scanning electron microscope (SEM, Ultra Plus, Carl Zeiss, Germany). For FB@S and FTGB@S samples, an ion sputtering apparatus was used tolectroplate samples in gold for subsequent electron microscopy (Fig. 3B). For hBMSCs samples, the cells were placed on a cell culture plate in advance and then sterilized with 75 % ethanol and ultraviolet lamps. Subsequently, hBMSCs were directly inoculated into a culture plate containing cell slides, cultured for 3 d, then removed from the medium, and washed with PBS three times for 3 min each time. Further, hBMSCs were fixed using 2.5 % glutaraldehyde. Finally, the cell slivers containing hBMSCs were placed on the conductive adhesive and fixed on the SEM stage for observation (Fig. S1).

### 2.6 Nanoscale mechanical and biomechanical testing

*Preparation of specimens:* All animal experiments were performed following the guidelines for the care and use of laboratory animals established by the Affiliated Hospital of Integrated Traditional Chinese and Western Medicine, Nanjing University of Chinese Medicine(Fig. S2). The protocol was approved by the Committee on the Ethics of Animal Experiments of the Hospital (Ethical Lot Number: 2022-LWKYS-046). The experimental procedures were approved by the hospital ethics committee. Ten New Zealand white female skeletally mature rabbits (2.5–3 kg, six months old) were euthanized for biomechanical testing. The lumbar motion segments of L1-L7 without posterior elements (vertebral body-IVD-vertebral body) were isolated and grouped by blocked randomization. Four groups (n = 4, biological replicates per group), which include Intact control, Un-repair control, FB@S, and FTGB@S were used for AFM testing. Six groups (n = 4, biological replicates per group), which include Intact control, Un-repair control, FB@S, FTGB@S, FB@S@F and FTGB@S@F were used for MTS testing (Fig. 3A). A hole punch was used to punch a hole (2 mm in diameter and 4 mm deep). 30 μl FTGB and FB hydrogels were injected into the hole of different groups, respectively, and a scaffold of the same size as the hole was removed from the acellular AF scaffold and implanted in the hole. In groups of FB@S@F and FTGB@S@F, a piece of subcutaneous fascia was removed and glued to the surface of the defect. It was pressed and left undisturbed for 5 min.

*Atomic force microscopy (AFM):* In AFM testing, twenty-four lumbar motion segments of L1-L7 without posterior elements were placed in medical gauze soaked with PBS and incubated in a water bath at room temperature for 4 h. The motion segments were cryosectioned in the sagittal plane (20 μm thick) using Kawamoto's film method [17]. After thawing the sections in PBS with protease inhibitors, AFM-nanoindentation was performed on defections using microspherical colloidal tips (AC240TM-R3–10, Olympus) and a dimension icon atomic force microscope (MFP 3D, America). The AC tapping mode was introduced to image the corresponding morphological characteristics in the air using a silicon nitride AFM tip with a curvature radius of 7 nm. Force spectroscopy was performed using a “closed loop” AFM system with a sensor piezo. Before acquiring force spectroscopy data, the AFM cantilever spring constant was calibrated through resonance frequency changes, which were induced by a small mass. The cantilever spring constant of the tips used in this study was calibrated as 0.1 N/m (Fig. 3C). As previously described, the modulus of the sample was calculated using the Hertz model [26].

*Mechanical testing & simulation (MTS):* In MTS testing, thirty-six lumbar motion segments of L1-L7 without posterior elements were placed in a water bath for 12 h at room temperature in medical gauze that was soaked with phosphate-buffered saline (PBS) (Fig. 3H). A biomechanical test was conducted, which included 20 sawtooth cycles (first 19 cycles of preconditioning, and the final cycle was used for analysis) of compression at −42 N at 0.5 Hz, followed by a 10-min creep test at −42 N, and a maximum failure stress test, which was performed as previously described [27] using an MTS Criterion Test system (MTS, Model 42, USA). Compressive modulus and compressive ROM were calculated, referring to representative force-displacement curves of motion segments in compression as previously described [28].

### 2.7 Assessment of the efficacy and mechanisms of *in vivo* repair

*Surgery:* Sixteen New Zealand White female skeletally mature rabbits (2.5–3 kg, six months old) were purchased for *in vivo* experiments. They were housed in separate cages under standard conditions. All animals were provided daily ad libitum access to tap water and food pellets. The anesthesia protocol for the other 16 rabbits that underwent surgery was based on the guidelines developed by the Institutional Animal Care Use Committee. After placing the rabbits in a lateral prone position, an 8–10 cm longitudinal skin incision was made, and dissection was continued down through the intermuscular plane between the paraspinal and external abdominal oblique musculature without damaging the peritoneum. The discs between the L2 and L6 vertebral bodies were exposed and grouped by blocked randomization. The molding method was the same as described in *2.6*. Finally, the wound was sutured layer by layer, and 40 w units of penicillin sodium were injected into the thigh muscle for prophylactic anti-infection treatment three days after surgery. All rabbits underwent MRI examinations and were sacrificed 12 w after the operation. The lumbar motion segments of L2-L6 without posterior elements (vertebral body-IVD-vertebral body) were isolated to perform histological, transcriptomics, immunohistochemical, and qPCR assays (Fig. S3).

*Magnetic resonance imaging (MRI):* All MRI scans were performed at 3.0 T (GE Discovery 3.0 T 750 W, America) on the L2-L6 discs. The NP area was calculated in the middle axial position of the disc on T2-weighted 3D images by the ImageJ software (version 2.3.0). The anterior disc height (Ha), middle disc height (Hm), and posterior disc height (Hp) were measured and calibrated as described by Sudo et al. [29]. According to a study by Seki et al. [29], the disc height (DH) was calculated as (Ha + Hm + Hp)/3. The disc height index (DHI) was defined as the DH of the disc in the Un-repair control, FB@S@F, or FTGB@S@F group/DH of the intact group. Percentage DHI (%DHI) was used to express the change in DHI: % DHI = (DHI of Un-repair control, FB@S@F, or FTGB@S@F group/baseline DHI) × 100 %. Three graders (two experienced and one inexperienced), blinded to the study groups, followed a modified Thompson classification system based on changes in the degree and area of signal intensity from grades 1 to 4 (1 = normal, 2 = minimal decrease in signal intensity but obvious narrowing of high-signal area, 3 = moderate decrease in signal intensity, and 4 = severe decrease in signal intensity) to evaluate the MRI data.

*Transcriptomics:* The samples were processed with TRIzol Reagent (INV, America). Subsequently, the experiments were independently conducted three times for each group. RNA sequencing was carried out by Lianchuan Biotechnology Co., Ltd (China). The gene expression levels of different groups were depicted through a volcanic map. In essence, differential expression analysis was executed using DESeq2 (v1.4.5), with a Q value set at ≤ 0.05. Moreover, heat map clustering analysis based on GO was analyzed in OmicStudio. Furthermore, the significance levels of terms and pathways were corrected using Bonferroni's correction, adhering to a strict Q-value threshold (Q-value ≤0.05).

*Histological assay:* The disc was split in two in the center of the defect, clearly visible where the arrow points (Fig. S5). The L2-L6 lumbar motion segments without posterior elements were isolated for histological assay (Fig. 5A). The sample sections (7 μm thick) were stained with the Hematoxylin and Eosin stain (H&E, Bios Europe, Skelmersdale, UK) and the Picrosirius Red stain (ab150681, Abcam, USA). Then, immunohistochemical (IHC) analyses of collagen I (COL1A1) (1:200, bsm-33400M, Bioss, China), collagen II (COL2A1) (1:200, bsm-33409M, Bioss, China), aggrecan (1:200, sc-33695, Santa Cruz, USA), mechanical stimulation signal pathway-related protein RhoA (1:100, sc-418, Santa Cruz, USA), ROCK (1:200, sc-19974, Santa Cruz, USA), and nerve ingrowth protein GAP 43 (1:50, sc-17790, Santa Cruz, USA) were performed. A negative control group was set up for all IHC samples. The specific steps were as follows:

The sample slide obtained by paraffin sectioning was treated with sodium citrate (P0083, Beyotime, China) for thermal induction to extract antigen. Then, the slide was treated with hydrogen peroxide solution (3 %) and incubated in the dark at room temperature for 25 min to block endogenous peroxidase. A buffer solution (10 % normal goat serum and 0.3 % Triton X-100 in PBS) was used for infiltration and sealing. Next, the slides were incubated with primary antibodies (COL1A1, COL2A1, aggrecan, RhoA, ROCK and GAP43) overnight in a refrigerator at 4 °C. The slices were washed with PBS (pH 7.40, 0.01M) thrice, and after 5 min each time, the slices were co-incubated with the corresponding secondary antibodies (HRP-conjugated goat anti-mouse, Proteintech, 1:100 dilution) again in the dark at 37 °C for 2 h. Then, the DAB (ZLI-9018, ZSGB-BIO, China) chromogenic process was performed as follows. The sections were placed in a PBS solution and washed thrice in a shaker for 5 min each time. Next, the sections were slightly dried, and the DAB developer solution was added. The color development time was controlled by viewing under the microscope; the positive color was brown and yellow. Then, the sections were washed with tap water to stop the development of color, and then they were re-stained with hematoxylin. Finally, the sections were observed under a fluorescence microscope. The histological score was graded by three graders (two experienced and one inexperienced), blinded to the study groups. The histological score of the degeneration of AF, NP, endplate, and IVD included eight categories, according to the previous study [30]. The histological score ranged from a normal morphology with 0 points to a severe degenerative change with 2 points. The migration and distribution of cells and the morphology of AF were observed in H&E and picrosirius red staining.

*Quantitative real-time PCR (qPCR) analysis:* The total RNA of the defects of twenty-four L2-L6 lumbar motion segments was isolated using Trizol reagents (15596026, Invitrogen, USA) following the manufacturer's instructions. A reverse transcription kit (E6560, NEB, USA) was used to synthesize cDNA from the RNA, followed by qPCR analysis using SYBR Premix (E3003, NEB, USA) and ABI STEPONE PLUS. The expression of the COL 1, COL 2, Aggrecan, RhoA, ROCK, Sox9, and GAP43 genes were analyzed by qPCR assays. Glyceraldehyde 3-phosphate dehydrogenase (GAPDH) was used as the reference gene. The sequences of the genes are shown in Table 2.

**Table 2:** The primer sequences used for the qPCR analysis.

Name		5→sequence→3	Product size	NCBI Reference Sequences (Ref Seq)
GAPDH	Sense	CGTGAACCACGAGAAGTATGA	568	NM 001082253.1
	Antisense	CCTGTTGCTGTAGCCAAATTC		
COL 1	Sense	CGTGAACCACGAGAAGTATGA	985	XM_017348831.1
	Antisense	CCTGTTGCTGTAGCCAAATTC		
COL 2	Sense	TCCTGTGCGACGACATAATC	392	NM_001195671.1
	Antisense	TCCAGCCTTCTCGTCAAATC		
Aggrecan	Sense	GCACCAGTGGAGCTGATATT	399	XM_008251722.2
		CAGACCCTAAGCCTTCTTCTTC		
RhoA	Sense	ACTGGTGATTGTCGGTGATG	248	XM_002713428.3
	Antisense	CAGGGCTGTCAATGGAGAAA		
ROCK	Sense	TTTCGGACCTTTCGGATTCTA	537	NM_001082367.1
	Antisense	AGTGATCTCTGTGGCACTTAAC		
SOX9	Sense	GAGAGCGAAGAGGACAAGTTC	329	XM_008271763.2
	Antisense	TAGTCCGGGTGGTCTTTCT		
GAP43	Sense	CCCGAAGACAAAGCTCACAA	607	XM 008266894.2
	Antisense	CGCCTTCTTCTTTACCCTCATC		

### 2.8 Statistical analysis

All quantitative data were expressed as the mean ± standard deviation (SD). The differences between the groups were determined by paired Student's t-tests, and the differences among multiple groups were determined by one-way analysis of variance (ANOVA). All differences were considered to be statistically significant at P < 0.05 (∗P < 0.05 and ∗∗P < 0.01). All tests were performed using GraphPad Prism 6.0 (GraphPad Software, La Jolla, CA, USA) and Origin 2022 (OriginLab Corporation, Northampton, MA, USA).

## 3 Results

### 3.1 Structural characterization and biocompatibility analysis

The FTIR spectra were performed to analyze the composition of FTG hydrogel (Fig. 2B). **Fibrinogen:** A distinct broad peak is observed near 3300 cm^−1^, attributable to the stretching vibrations of the N-H group in fibrinogen, which overlaps with O-H stretching vibrations due to residual water. The absorption peak at 1640 cm^−1^ corresponds to the C=O stretching vibration of the protein backbone (amide I). The absorption peak near 1570 cm^−1^ is attributed to the N-H bending vibration and C-N stretching vibrations (amide II). A bending vibration absorption peak from the N-H group is observed around 1390 cm^−1^, potentially corresponding to amide III [31]. **Thrombin:** The C=O stretching vibration of the polypeptide backbone is observed at 1634 cm^−1^ (amide I). A peak near 3350 cm^−1^ is primarily assigned to the N-H stretching vibration, likely overlapping with O-H stretching vibrations. This suggests the presence of intramolecular hydrogen bonding within the protein structure [32]. **Genipin:** The structure of genipin contains numerous hydroxyl groups, resulting in a broad and prominent peak near 3300 cm^−1^ due to O-H stretching vibrations. The carbonyl group, a distinguishing feature of the genipin molecule, exhibits a stretching vibration absorption peak at 1680 cm^−1^. Additionally, the stretching vibration of the conjugated C=C double bond in the molecule appears as an absorption peak around 1625 cm^−1^, characteristic of the α,β-unsaturated lactone structure. An ether bond (C-O) stretching vibration absorption peak is observed near 1195 cm^−1^, indicative of the lactone ring structure. **FTG:** A broad peak near 3360 cm^−1^, corresponding to N-H stretching vibrations from proteins and overlapping with O-H stretching vibrations from genipin and residual water. This broadening suggests intermolecular hydrogen bonding and potential crosslinking interactions. A peak at 1644 cm^−1^, primarily attributed to the C=O stretching vibration (amide I) of fibrinogen and thrombin, with a slight shift indicating new interactions due to crosslinking. An absorption peak near 1560 cm^−1^, assigned to the C-N stretching and N-H bending vibrations (amide II), reflecting the formation of secondary amides resulting from the reaction of genipin with fibrinogen and thrombin. A peak around 1380 cm^−1^, corresponding to the in-plane bending vibration of the N-H group (amide III), further supporting the formation of crosslinked amide structures. A vibration absorption peak at 1017 cm^−1^, attributed to C-O stretching vibrations, likely arising from residual hydroxyl or ester groups in the genipin and protein complex. The FTIR spectrum of FTG demonstrates characteristic peaks from all components, confirming successful crosslinking [33].

Furthermore, we performed biocompatibility analysis. As shown in Fig. 1C, compared with FB group, FTGB group showed more calcein AM staining areas and less calcein PI staining areas. Cell viability in FTGB group was higher than that in FB group (97.60 ± 2.02 *vs* 81.43 ± 4.50) (P < 0.01) (Fig. 2F). As shown in Fig. 2D, compared with FB group, FTGB group showed more DAPI staining and Edu staining areas. Quantitative statistical analysis also showed that the number of nuclei and proliferating cells in FTGB group was significantly higher than that in FB group (P < 0.01) (Fig. 2G). Furthermore, compared with FB group, FTGB group showed more phalloidine staining areas (Fig. 2I). Mean fluorescence density of phalloidine in FTGB group was significantly larger than that in FB group (P < 0.01) (Fig. 2H). In summary, FTGB hydrogel has good biocompatibility and can be competent for subsequent *in vivo* experiments.

### 3.2 Nanoscale mechanical and biomechanical analysis

The structure of FTGB@S is reticular, in which hydrogels, hBMSCs cells and acellular scaffolds interweave and interact with each other to form matrix. Refer to SEM image of hBMSCs (Fig. S1), the distribution of hBMSCs cells in the hydrogel also shows a similar colony shape, which fills the gap of the original acellular scaffold, and helps to improve the overall performance of the material as an enhancement phase (Fig. 3B). In contrast, the structure of FB@S shows loose fault structure with more voids, and the distribution of hBMSCs is relatively dispersed. The results showed that FTGB@S showed better performance and the potential to regulate cell growth. The microstructure of the Un-repaired and the FB@S groups was loose and disordered, while that of the FTGB@S group was tightly ordered and similar to that of the Intact control group (Fig. 3C). Further, compared with the Intact control, the sample modulus of outer AF (53.58 ± 8.94 *vs* 10.74 ± 1.33 Mpa), inner AF (41.23 ± 7.28 *vs* 9.99 ± 1.03 Mpa) and NP (44.82 ± 9.88 *vs* 27.64 ± 17.69 Mpa) in the FTGB@S group performed better. (P < 0.01) (Fig. 3D–F). Therefore, the results showed that FTGB@S can help the damaged AF restore its microstructure and improve its elastic modulus.

In the axial compression test, the FTGB@S@F group resisted the cyclic axial load (25.53 ± 1.17 Mpa) and effectively maintained the disc height (0.57 ± 0.12 mm), which was the best in these groups (Fig. 3H–J). The constant 42 N creep experiment results showed that the FTGB@S@F group could resist the daily load for a long time and produce small deformation, which was close to Intact control, reflecting stable axial compression resistance (Fig. 3K and L). The maximum failure stress test results showed that the FTGB@S@F group had the lowest rupture ROM (1.45 ± 0.17 mm) among all groups, and its rupture modulus ranked only second to the Intact control (1.34 ± 0.36 *vs* 1.51 ± 0.45 Mpa) (Fig. 3M and N). Therefore, the results showed that FTGB@S@F can help the damaged AF recover its mechanical function and resist the load of daily activities.

### 3.3 Magnetic resonance imaging (MRI) assay

**Fig. 4:**
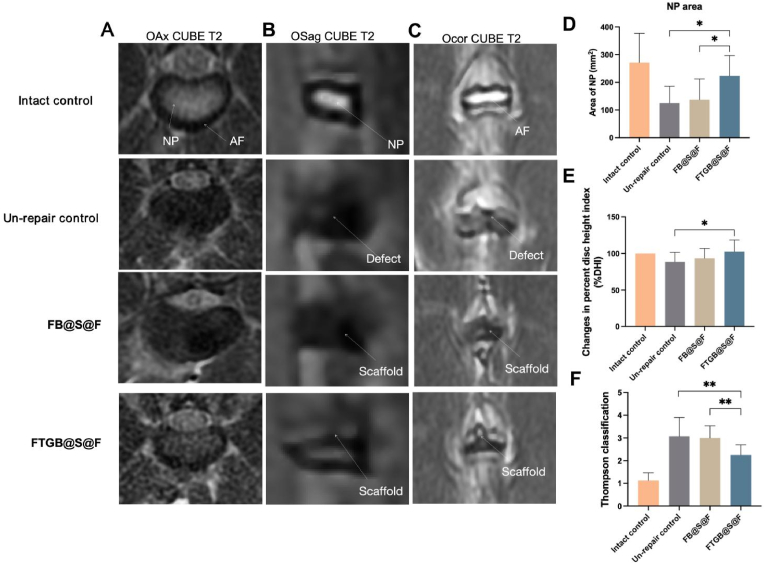
MRI evaluation. (A–C) MRI images of rabbit intervertebral discs at 12 weeks post-modeling. “OAx,” “Osag,” and “Ocor” represent axial, sagittal, and coronal views, respectively. (D) The NP area of different interventions. (E) Changes in the percent disc height index (%DHI) of different interventions. (F) Thompson's classification of different interventions. The error bars indicate SD. n = 4 (∗P < 0.05 and ∗∗P < 0.01).

The characterization of the samples in the FTGB@S@F group based on MRI examinations showed that the AF structure was relatively complete, and the scaffold stayed in position, showing a high signal intensity (Fig. 4A–C). In contrast, the AF structure in the Un-repair control and the FB@S@F group was considerably damaged, and the NP region showed a low signal intensity. The NP area of FTGB@S@F group (223.64 ± 73.32 mm^2^) was significantly larger than that of FB@S@F group (137.30 ± 75.31 mm^2^) (P < 0.05) (Fig. 4D). Changes in the percent disc height index indicated that the height of the discs in the FTGB@S@F group was significantly higher than that in the Un-repair control (102.5 ± 73.32 % *vs* 88.50 ± 12.86 %) (P < 0.05) (Fig. 4E). According to the Thompson classification, degeneration in the FTGB@S@F group was significantly less severe than that in the Un-repair control and the FB@S@F group (P < 0.01) (Fig. 4F). When comparing segmental repair outcomes, the NP area, disc height, and degree of disc degeneration in the Un-repair control and FB@S@F groups exhibited a general decline with progression to lower lumbar segments. This suggests that injury-induced degeneration is more severe in the lower lumbar spine, with a corresponding increase in repair difficulty. In contrast, the FTGB@S@F group maintained relatively stable repair performance across all segments, demonstrating particularly superior outcomes in lower lumbar disc height and degeneration indices, which were significantly better than those observed in the other two groups (Fig. S4).

**Fig. 5:**
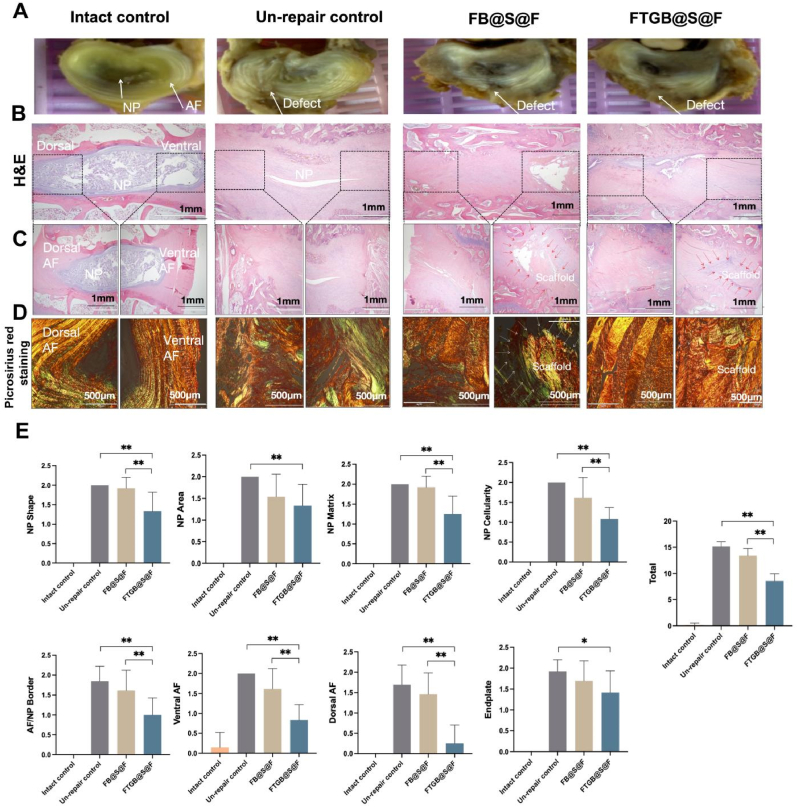
Hematoxylin and Eosin (H&E) staining, picrosirius red staining, and immunohistochemical staining. (A) Specimen images of rabbit intervertebral disc at 12 w post-modeling. (B) The central region of the histological sections in H&E staining. (C) Dorsal and ventral AF in H&E staining (Left: dorsal AF, right: ventral AF). (D) Dorsal and ventral AF in picrosirius red staining. (E) The histological score of different interventions. The error bars indicate SD. n = 4 (∗P < 0.05 and ∗∗P < 0.01).

### 3.4 Histological analysis

The disc was split in two in the center of the defect, clearly visible where the arrow points (Fig. S5). The images showed that AF structure was disordered in the Un-repaired group, and NP degeneration was obvious. In the FB@S@F group, the damaged AF did not heal well, and the NP deteriorated to some extent. In FTGB@S@F group, the structure of the damaged AF was relatively orderly, and the degeneration of the NP was not obvious (Fig. 5A). Further, in the Un-repair control, the staining of samples with H&E and the picrosirius red staining showed a disordered internal disc structure and severe destruction of the NP and AF, while the degree of disc degeneration was less severe in the FB@S@F group and the FTGB@S@F group, and the internal structure of IVD was more complete (Fig. 5B–D). The scaffold could be found in those groups where the red arrow points. However, the cross-section connection in the FB@S@F group was not tight, the structure of the new organization is chaotic and loose, and the contralateral AF was partly damaged. In contrast, the defect was filled with continuous and ordered collagen fibers in the FTGB@S@F group, and the contralateral AF was preserved. The histological score, which included the NP shape, NP matrix, NP cellularity, AF/NP border, and the AF of the FTGB@S@F group, was also significantly lower than those in the un-repair group and the FB@S@F group (P < 0.01) (Fig. 5E). In the segmental repair analysis, the degree of intervertebral disc and ventral annulus degeneration at L4-L5 and L5-L6 in the Un-repair and FB@S@F groups was greater than at L2-L3 and L3-L4, exhibiting an overall increasing trend. This indicates that as daily stress on the intervertebral disc and annulus intensifies, the difficulty of repair progressively increases. Furthermore, the higher stress in the Un-repair and FB@S@F groups may accelerate disc degeneration and exacerbate the original AF injury. In contrast, the FTGB@S@F group demonstrated superior repair effects in the lower lumbar segments, suggesting that controlled stimulation from daily stress may enhance its repair efficacy. Compared to the other groups, FTGB@S@F effectively delayed intervertebral disc degeneration, facilitated ventral AF repair, and provided protection for the dorsal AF (Fig. S6).

### 3.5 Transcriptomics analysis

**Fig. 6:**
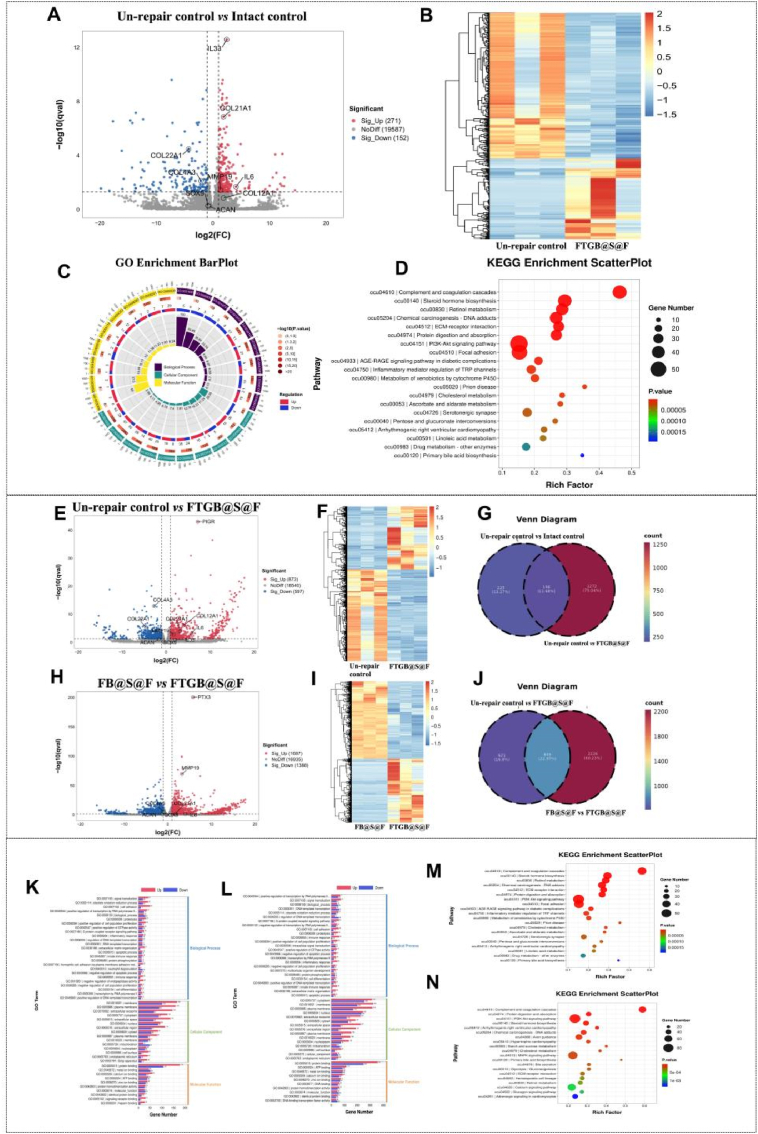
Transcriptomics analysis of rabbit intervertebral disc at 12 w post-modeling. (A) Volcano plot of significantly altered genes from RNA-seq analysis on the Un-repair control and Intact control. (B) Hierarchical clustering of transcriptomic signature from Un-repair control and Intact control. (C) GO enrichment barPlot of Un-repair control *vs* Intact control. (D) KEGG enrichment scatterplot of Un-repair control *vs* FTGB@S@F group. (E) Volcano plot of significantly altered genes from RNA-seq analysis on the Un-repair control and FTGB@S@F group. (F) Hierarchical clustering of transcriptomic signature from Un-repair control and FTGB@S@F group. (G) GO enrichment barPlot of Un-repair control *vs* FTGB@S@F group. (H) KEGG enrichment scatterplot of Un-repair control *vs* FTGB@S@F group. (I) Venn diagram of differentially expressed genes between Un-repair control *vs* Intact control and Un-repair control *vs* FTGB@S@F group. (J) Volcano plot of significantly altered genes from RNA-seq analysis on FB@S@F group and FTGB@S@F group. (K) Hierarchical clustering of transcriptomic signature from FB@S@F group and FTGB@S@F group. (L) GO enrichment barPlot of FB@S@F group *vs* FTGB@S@F group. (M) KEGG enrichment scatterplot of FB@S@F group *vs* FTGB@S@F group. (N) Venn diagram of differentially expressed genes between Un-repair control *vs* FTGB@S@F group and FB@S@F group *vs* FTGB@S@F group. n = 3.

To reveal the underlying mechanism, RNA transcriptome sequencing was performed and analyzed. Volcano plot and hierarchical clustering of significantly altered genes from RNA-seq analysis of the Un-repair control *vs* Intact control showing differential clustering of associated genes revealed broad gene expression differences with the upregulation of IL33 and COL21A1 genes and the downregulation of the COL22A1 gene (Fig. 6A and B). Through Gene Ontology (GO) analysis, differentially expressed genes were enriched in plasma membrane, membrane and protein binding (Fig. 6C). Through Genes and Genomes (KEGG) analysis, differentially expressed genes were enriched in PI3K-Akt signaling pathway and Focal adhesion (Fig. 6D). By contrast, volcano plot and hierarchical clustering of significantly altered genes from RNA-seq analysis of the Un-repair control *vs* FTGB@S@F group revealed broad gene expression differences with PIGR gene upregulated and COL4A3 gene downregulated (Fig. 6E and F). Through GO analysis, differentially expressed genes were enriched in protein binding, membrane and plasma membrane (Fig. 6K). Through KEGG analysis, differentially expressed genes were also enriched in PI3K-Akt signaling pathway and Focal adhesion (Fig. 6M). Venn diagram of differentially expressed genes between the Un-repair control vs Intact control and the Un-repair control *vs* FTGB@S@F group showed that the proportion of common differentially expressed genes was 11.68 % (Fig. 6G). Volcano plot and hierarchical clustering of significantly altered genes from RNA-seq analysis of the FB@S@F *vs* FTGB@S@F group revealed broad gene expression differences with PTX3 and MMP19 genes upregulated and COL4A3 gene downregulated (Fig. 6H and I). Through GO analysis, differentially expressed genes were enriched in protein binding, membrane and cytoplasm (Fig. 6L). Through Kyoto Encyclopedia of KEGG analysis, differentially expressed genes were also enriched in the PI3K-Akt signaling pathway and MAPK signaling pathway (Fig. 6N). Venn diagram of differentially expressed genes between the Un-repair control vs FTGB@S@F group and the FB@S@F *vs* FTGB@S@F group showed that the proportion of common differentially expressed genes was 22.97 % (Fig. 6J).

### 3.6 Immunohistochemical staining analysis

**Fig. 7:**
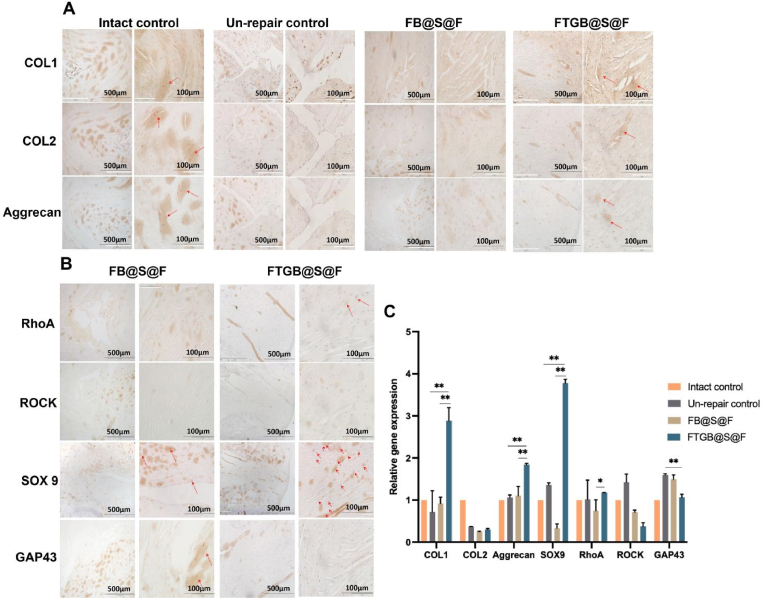
Immunohistochemical staining and qPCR analysis of rabbit intervertebral disc at 12 w post-modeling. (A) Immunohistochemical staining of COL 1, COL2, and Aggrecan (The red arrow indicates the positive area). (B) Immunohistochemical staining of RhoA, ROCK, SOX 9 and GAP 43. (C) The qPCR analyses for the relative expression of genes (COL 1, Aggrecan, SOX 9 and RhoA). The error bars indicate SD. n = 4 (∗P < 0.05 and ∗∗P < 0.01).

The expression levels of key proteins COL 1, COL 2 and Aggrecan of AF regeneration in FTGB@S@F group were significantly higher than those in Un-repair control and FB@S@F groups (Fig. 7A). Additionally, the expression levels of mechanical stimulation signaling pathway-related proteins RhoA and SOX-9 in FTGB@S@F group were significantly higher than those in FB@S@F group. In contrast, the expression levels of the neural growth protein GAP 43 in FTGB@S@F group was significantly lower than that in FB@S@F group (Fig. 7B).

### 3.7 Quantitative real-time PCR (qPCR) analysis

The results of the qPCR analysis showed similar results as those found for the immunohistochemical staining, i.e., the gene expression of COL 1, Aggrecan, SOX 9, and RhoA in the FTGB@S@F group was significantly higher than that in the un-repair group and the FB@S@F group (P < 0.05) (Fig. 7C). However, the gene expression of GAP 43 in the FTGB@S@F group was lower than that in the Un-repair control. Therefore, the results showed that the crosslinking stabilization strategy effectively activated the RhoA pathway, promoted the regeneration and repair of the damaged AF, and inhibited the growth of nerve fibers.

## 4 Discussion

**Fig. 8:**
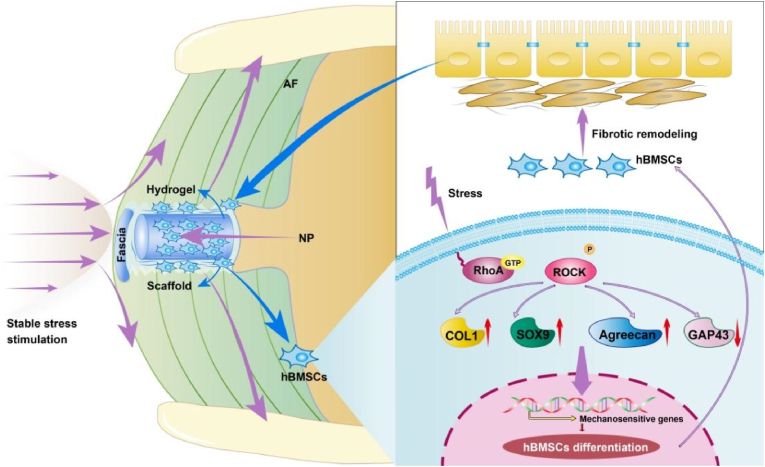
Schematic diagram of the crosslinking stabilization strategy and mechanism diagram of the crosslinking stabilization strategy promoting the differentiation of hBMSCs into fibrocycle-like cells.

Many studies have demonstrated that repairing annulus fibrosus (AF) damage is crucial for the effective treatment of intervertebral disc (IVD) diseases [34,35]. However, these studies primarily focus on restoring the fibrous annulus structure. It is equally important to provide a stable stress balance environment to promote orderly regeneration. Stress plays a pivotal role in this process, as the mechanical environment of the damaged AF is often chaotic (Fig. 1B). Therefore, we need to develop a mechanical guide that can ensure proper stimulation of the implanted stem cells by stress, facilitating the orderly regeneration of the damaged AF region. In this study, we proposed an innovative repair strategy combining hydrogel, seed cells, and scaffold to repair a rabbit AF injury model (Fig. 8). Biocompatibility tests showed that our method exhibited good compatibility. Macroscopic and microscopic mechanical experiments confirmed that our repair strategy significantly enhanced the mechanical load-bearing capacity and elastic modulus of the damaged disc, restoring the original stress transmission structure. Furthermore, MRI, immunohistochemical examinations, and qPCR analysis revealed that the crosslinking stabilization strategy promoted the differentiation of seed cells, restored the damaged AF structure, and facilitated its orderly regeneration. Our results emphasize that stress is a key factor in the AF repair process and is essential for the proper regeneration of damaged AF. Additionally, the crosslinking stabilization strategy demonstrated promising repair effects, laying the foundation for future clinical applications of tissue engineering in AF repair.

AF injury can lead to various adverse outcomes, including inflammation, reduction in disc height, and nucleus pulposus (NP) herniation. However, disruption of the mechanical environment is the most critical factor contributing to accelerated disc degeneration [36]. The maintenance of normal physiological function of the IVD relies on a stable mechanical environment to withstand complex external stresses, including compression, tension, and shear forces [37,38]. Physiological mechanical stress can induce anabolic activity, but when the original stress environment is disrupted, continuous mechanical overload can lead to catabolic events in NP cells [39], thereby exacerbating AF injury [40]. This decreases the disc's mechanical capacity and increases the risk of further damage. A stable mechanical environment is essential for effective AF regeneration. Human bone marrow-derived mesenchymal stem cells (hBMSCs), which serve as key seed cells for AF repair, are highly sensitive to mechanical cues that influence their differentiation. Recent study of how fluid viscosity affects the behavior of hBMSCs on viscoelastic substrates has deepened our understanding of how adhesion reshapes stem cell differentiation [41]. In our study, the crosslinked stabilization strategy does more than just provide the viscosity of extracellular fluid; it establishes a stable mechanical stimulation environment. This reveals the significant role of synergistic internal and external mechanical stability in guiding the orderly differentiation of stem cells. The RhoA/ROCK signaling pathway is central to hBMSCs differentiation under mechanical stimulation. In our prior work, we demonstrated that mechanical cues could regulate hBMSCs differentiation into AF-like cells through this pathway. Therefore, restoring the original stress conduction pathways and maintaining a stable mechanical environment are critical for promoting AF regeneration [8].

In this study, we developed an effective method for AF regeneration using genipin bio-glue to bond the cross-section, thereby restoring the original stress conduction pathway. This approach facilitated stable stress stimulation and allowed for the incorporation of an acellular scaffold to improve the mechanical load-bearing capacity and stability of the IVD. Our findings demonstrated that the acellular scaffold significantly enhanced the mechanical capacity of the AF. However, in our preliminary experiments, the acellular scaffold was easily dislodged from the defect due to high internal IVD pressure, leading to implantation failure. To address this issue, we developed a novel external fascial fixation technique for reinforcement, which significantly improved the mechanical stability of the repair model. The combination of fascia and bio-glue for external fixation yielded the expected results, enhancing the mechanical stability of the damaged disc and ensuring continuous and stable stress stimulation, which helped establish a conducive environment for internal repair. Transcriptome analysis further revealed that the PI3K-Akt signaling pathway and focal adhesion play significant roles in the physiological processes of disc injury and repair, promoting regeneration and reducing inflammatory responses. Comparison between the FB@S@F and FTGB@S@F groups showed increased activity in the PI3K-Akt and MAPK signaling pathways. As a key component of focal adhesions, the MAPK signaling pathway is instrumental in promoting focal adhesion and cytoskeletal interactions, while the PI3K-Akt signaling pathway supports cell survival and proliferation by preventing apoptosis through multiple mechanisms [42]. The RhoA/ROCK pathway, which acts as a bridge between focal adhesions and the cytoskeleton, is closely associated with these two pathways [43]. It facilitates bidirectional regulation between focal adhesions and cytoskeletal changes by promoting actin polymerization and participating in cytoskeletal stability. Therefore, we will focus on these pathways in our subsequent analysis. Our repair strategy was further validated by histochemical analysis, which demonstrated increased expression of COL 1, Aggrecan, SOX 9, and RhoA, suggesting that our strategy effectively promoted seed cell differentiation and facilitated the regeneration and repair of the damaged AF (Fig. 8).

Additionally, a study found that GAP43, a protein involved in nerve growth and regeneration, is associated with the pathogenesis of chronic lower back pain [44]. In patients with this condition, nerve growth into the affected IVD exacerbates discogenic low back pain. Previous studies have shown that chronic discogenic low back pain results from dynamic overloading and compression of the IVD, which leads to a sustained increase in inflammatory mediators, causing nerve damage and ingrowth of nerve fibers [45]. We found that GAP43 expression decreased after implementing our repair strategy, suggesting reduced nerve fiber growth and inhibition of discogenic lumbago. By restoring the stress stimulation environment of the IVD, we stabilized the stress environment and partially reduced dynamic overload, which led to decreased nerve fiber growth and improved repair outcomes. However, the underlying mechanisms need further investigation.

Although our results underscored the importance of stable mechanical stimulation for AF repair, our study had some limitations. The external fascial fixation method used in this study presented operational challenges and may be difficult to implement on a wider scale. Therefore, it is essential for future research to design a more convenient and stable method for restoring stress conduction that can be easily implanted. While our study focused on the role of stable stress stimulation in AF repair, other factors, such as inflammation and hydrogel adhesion, also significantly influence the repair process. Further research is needed to elucidate the contributions of these factors comprehensively. Designing more comprehensive experiments that consider the complex interactions among these factors will help us better understand the mechanisms underlying AF repair and develop more effective treatment strategies. Such studies will enhance our understanding of AF regeneration and contribute to the development of improved therapeutic approaches for patients with IVD disease.

## 5 Conclusion

Our study underscores the critical role of synergistic internal and external mechanical stability in driving the orderly differentiation of stem cells. Building on this insight, the crosslinked stabilization strategy we developed effectively restores the mechanical properties of damaged annulus fibrosus (AF) and promotes its orderly regeneration and repair. This approach lays a solid foundation for the future clinical application of tissue engineering in AF injury repair.

## CRediT authorship contribution statement

**Zihan Wang:** Writing – original draft, Visualization, Validation. **Lei Wang:** Formal analysis, Data curation. **Shaoshuo Li:** Data curation, Conceptualization. **Xin Chen:** Investigation, Funding acquisition. **Bo Chen:** Formal analysis, Data curation. **Zhichao Lou:** Funding acquisition, Formal analysis. **Zheng Li:** Investigation, Funding acquisition, Formal analysis. **Rongrong Deng:** Project administration, Methodology. **Lin Xie:** Data curation, Conceptualization. **Jianwei Wang:** Formal analysis, Conceptualization. **Xin Liu:** Writing – review & editing, Supervision, Resources, Conceptualization. **Ran Kang:** Data curation, Conceptualization.

## Ethics approval and consent to participate author-disclosure

All animal experiments were performed and approved at the Institute of Jiangsu Provincial Hospital of Integrative Chinese and Western Medicine (Nanjing, China) and adhered to the guidelines of the Committee for Research and Animal Ethics (Ethical Lot Number: 2022-LWKYS-046).

## Consent for publication author-disclosure

All authors have seen the manuscript and approved the submission.

## Declaration of competing interest

The authors declare that they have no known competing financial interests or personal relationships that could have appeared to influence the work reported in this paper.

## Data Availability

Data will be made available on request.
