# Supplementary material for: Crosslinking stabilization strategy: A novel approach to cartilage-like repair of annulus fibrosus (AF) defects
Source: Mater Today Bio. 2025 Mar 5;31:101625. doi: 10.1016/j.mtbio.2025.101625 (PMC11929887; doi:10.1016/j.mtbio.2025.101625)
Supplement: Multimedia component 1 [file mmc1.docx]

**Supporting Information**

## Crosslinking Stabilization Strategy: A Novel Approach to Cartilage-like Repair of Annulus Fibrosus (AF) Defects

Zihan Wang^1,2^, Lei Wang^1^, Shaoshuo Li^2^, Xin Chen^1,4^, Bo Chen^7^, Zhichao Lou^5^, Zheng Li^6^, Rongrong Deng^1,4^, Lin Xie^1^, Jianwei Wang^2*^, Xin Liu^1,4*^, Ran Kang^1,3*^

_______________________________________________________________________________

^1^Affiliated Hospital of Integrated Traditional Chinese and Western Medicine, Nanjing University of Chinese Medicine, Jiangsu Province 210028 P.R. China.

^2^Wuxi Hospital Affiliated to Nanjing University of Chinese Medicine, Jiangsu Province 214000 P.R. China.

^3^ Department of Orthopedics, Nanjing Lishui Hospital of Traditional Chinese Medicine, Nanjing, Jiangsu Province 210028 P.R. China.

^4^The Third Clinical Medical College, Nanjing University of Chinese Medicine, Nanjing, Jiangsu Province 210028 P.R. China.

^5^College of Materials Science and Engineering, Nanjing Forestry University, Nanjing, Jiangsu Province 210037 P.R. China.

^6^Peking Union Medical College Hospital, Beijing, 100730, P.R. China.

^7^Materials Science and Devices Institute, Suzhou University of Science and Technology, Suzhou 215009, P. R. China.

***Correspondence**: kangran126@126.com (**Ran Kang**); [liuxin@njucm.edu.cn](mailto:liuxin@njucm.edu.cn) (**Xin Liu**); wxzy006@njucm.edu.cn (**Jianwei Wang**)

_______________________________________________________________________________

**Supplemental Figures**


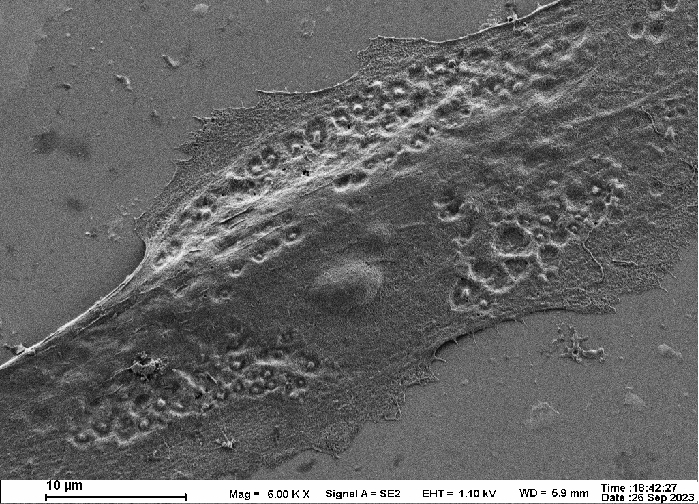


hBMSCs

**Figure S1.** Scanning electron microscope (SEM) image of hBMSCs (The white arrow refers to hBMSCs, which serve as a reference for identifying the morphology of stem cells within the hydrogel in Figure 3B).


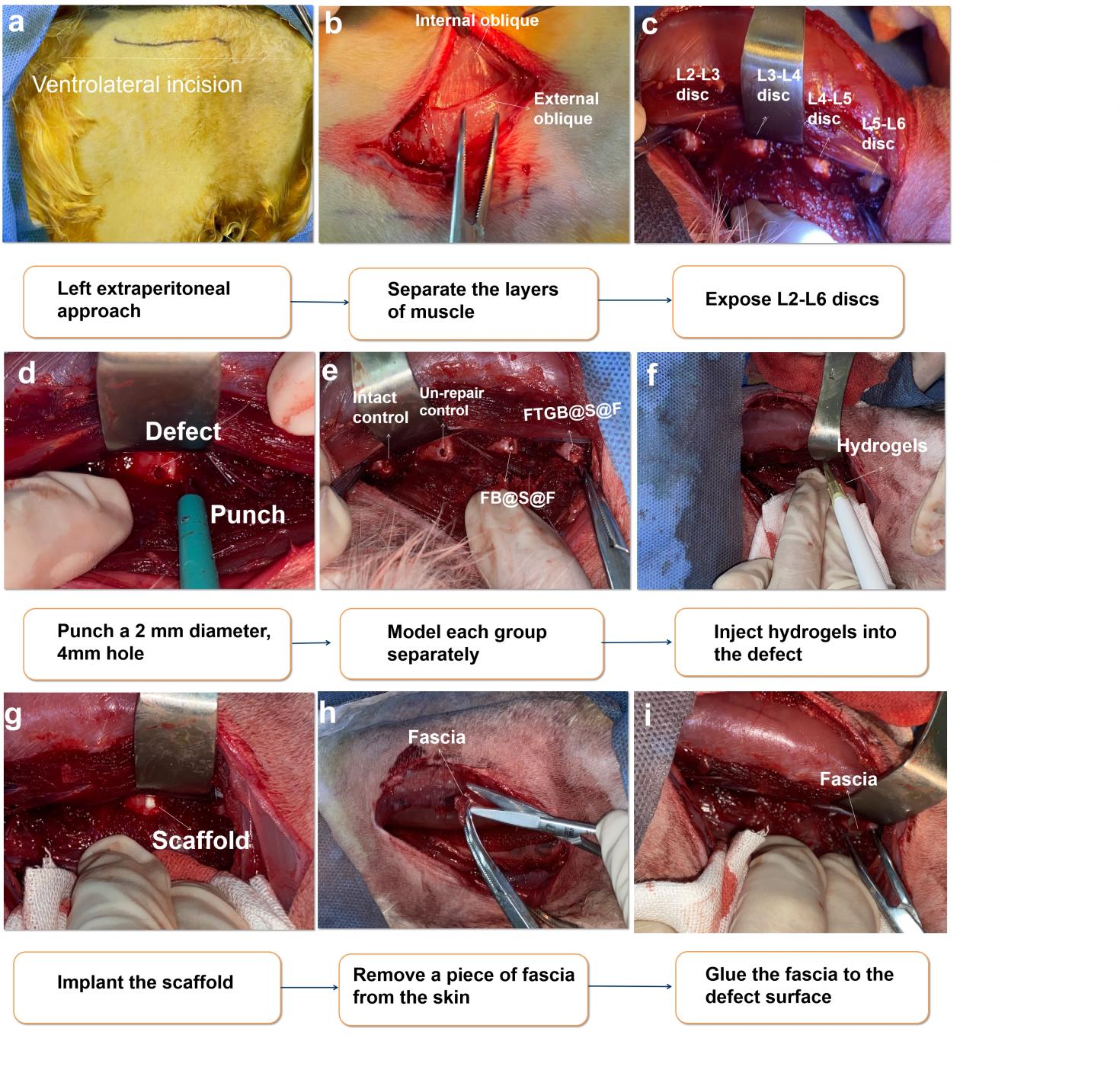


**Figure S2.** Diagram of the surgical procedure.


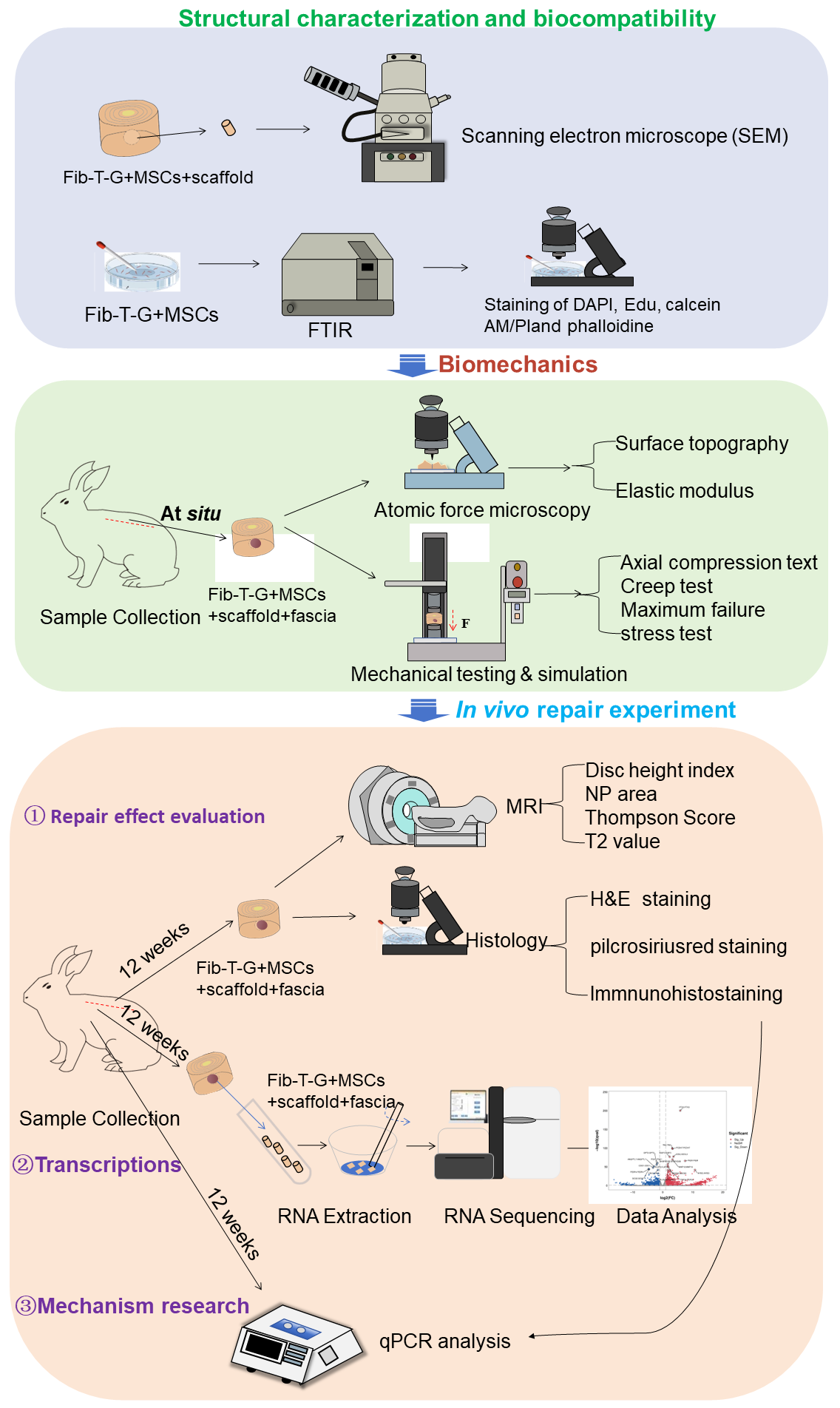


**Figure S3.** Schematic representation of the experimental design for assessing in vivo repair efficacy.


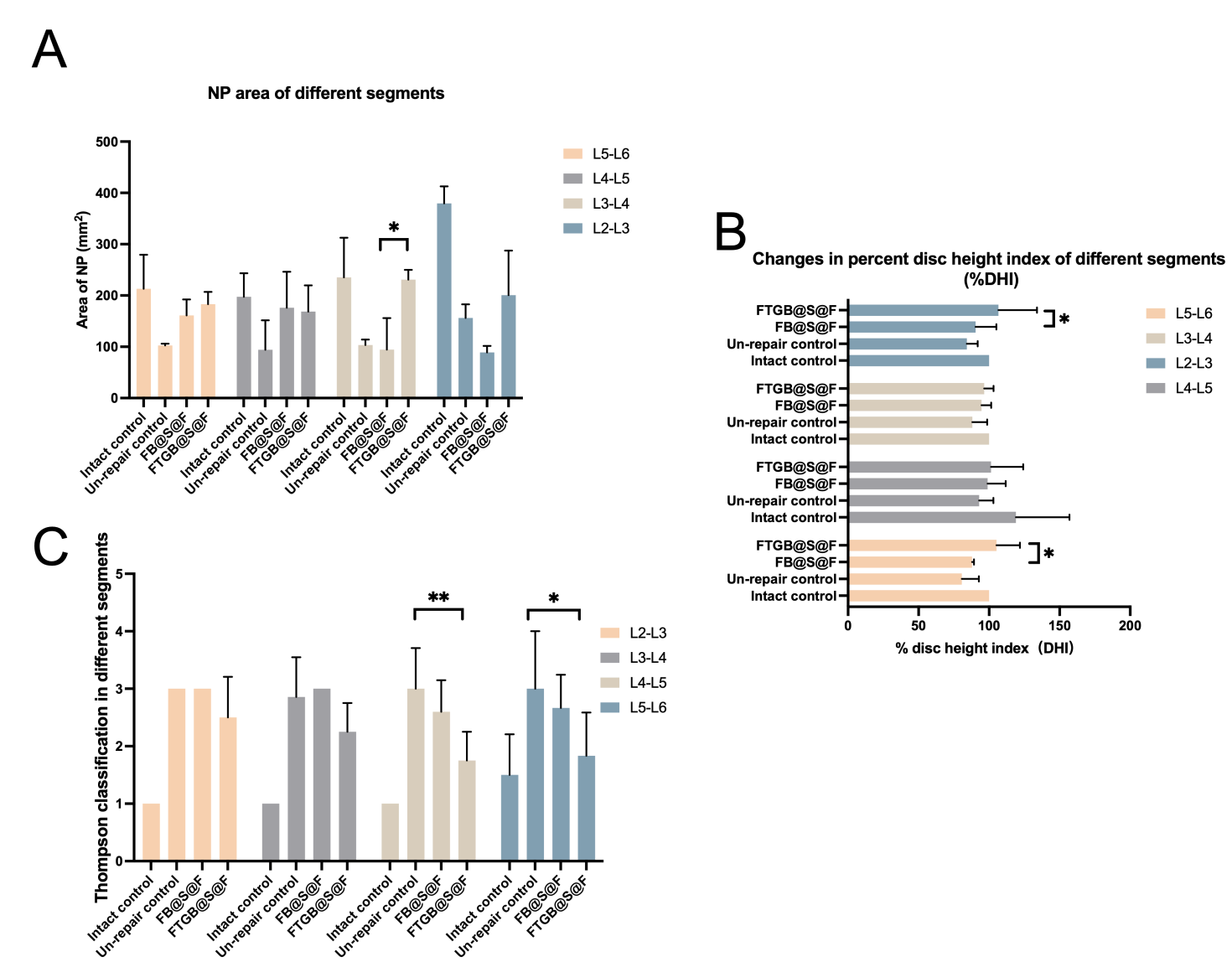


**Figure S4.** MRI evaluation. (A) NP area of different segmentss. (B) Changes in %DHI of different segments. (C) Thompson classification of different segments. The error bars indicate SD. n = 4 (*P < 0.05, **P < 0.01).


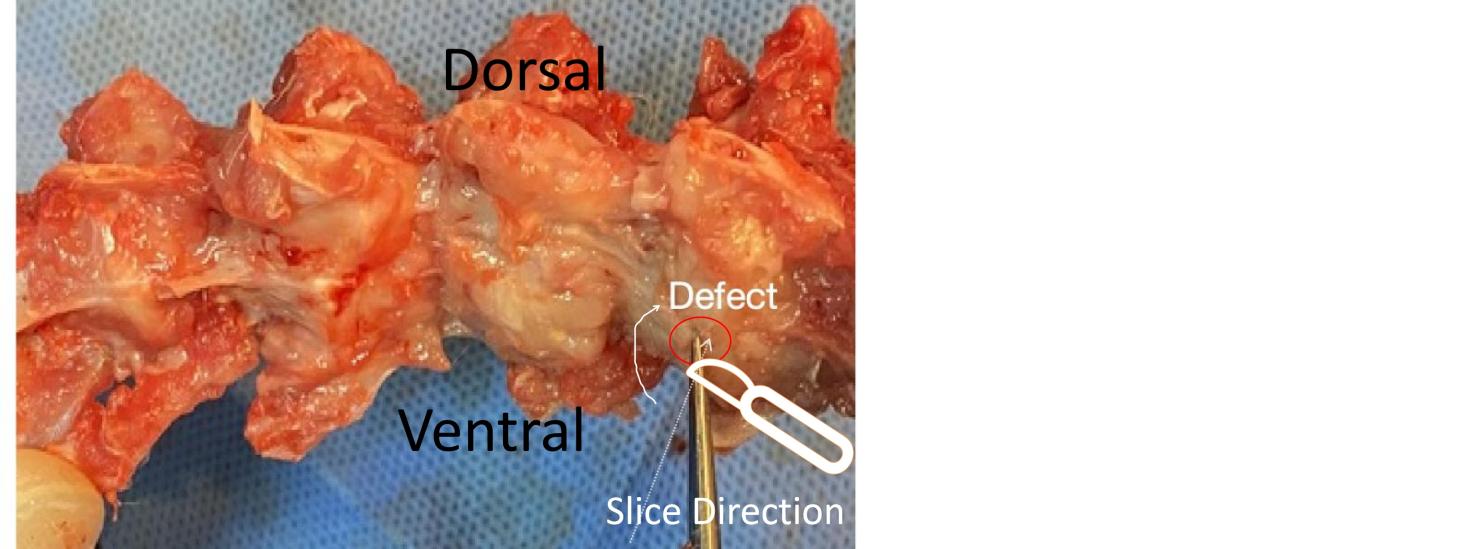


**Figure S5.** Diagram of slice direction.


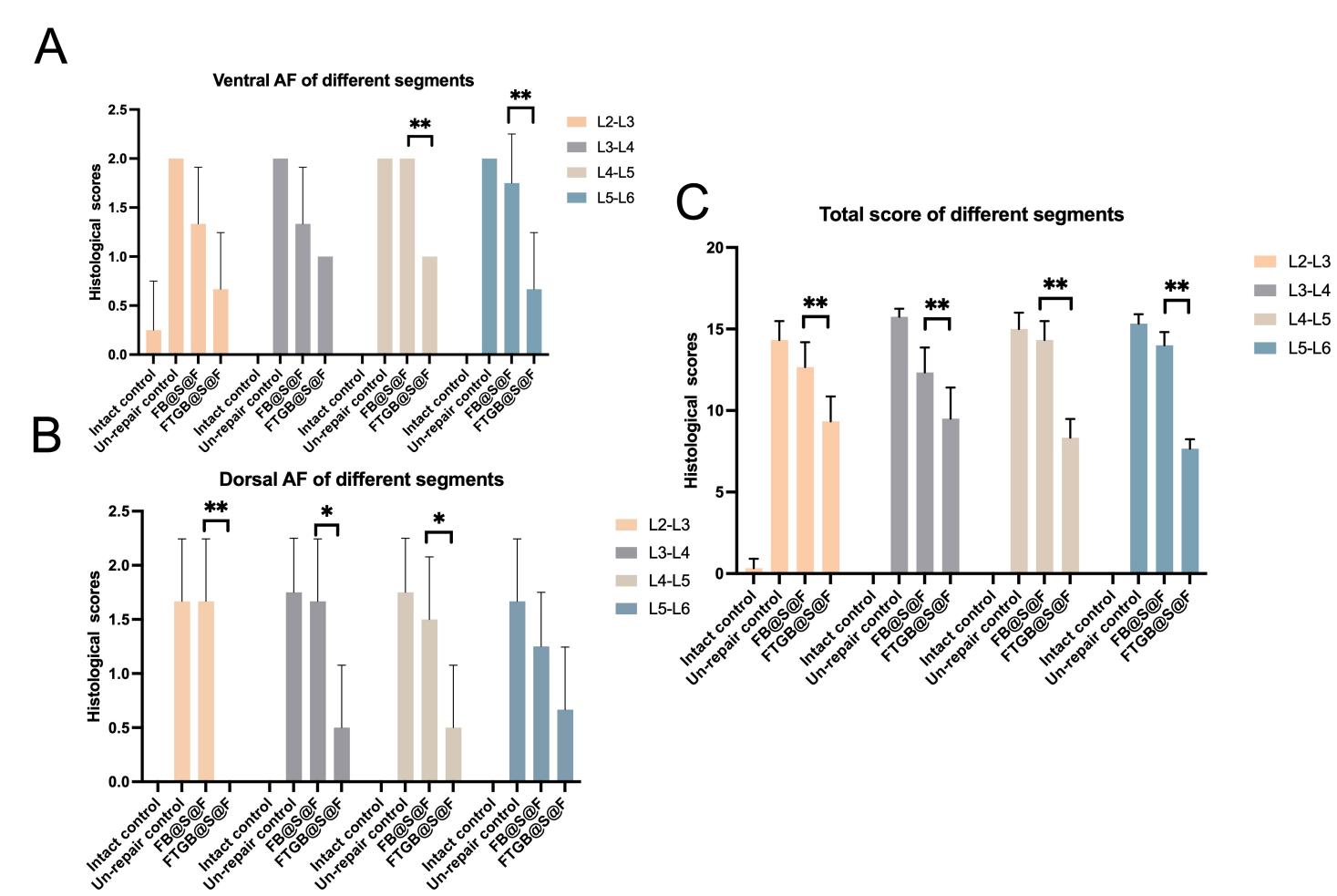


**Figure S6.** Immunohistochemical staining analysis. (A) Histological score of ventral AF at different segments. (B) Histological score of dorsal AF at different segments. (C) Histological score at different segments. The error bars indicate SD. n = 4 (*P < 0.05, **P < 0.01).


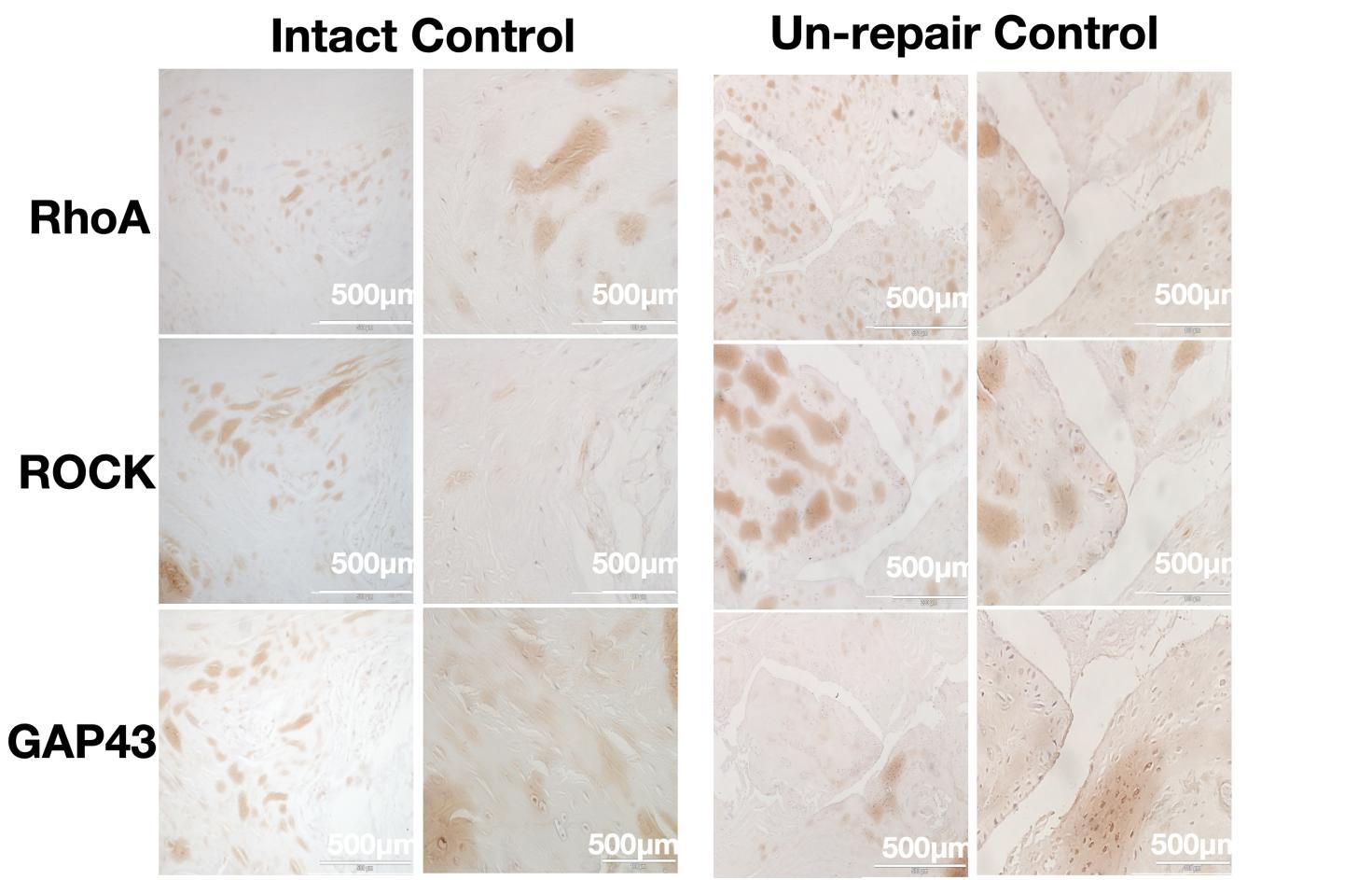


**Figure S7.** Immunohistochemical staining analysis. Immunohistochemical staining of RhoA, ROCK and GAP 43 in Intact control and Un-repair control.
